# An Evolutionary Model of Cooperation, Fairness and Altruistic Punishment in Public Good Games

**DOI:** 10.1371/journal.pone.0077041

**Published:** 2013-11-19

**Authors:** Moritz Hetzer, Didier Sornette

**Affiliations:** 1 Chair of Entrepreneurial Risks, Department of Management, Technology and Economics, ETH-Zurich, Zurich, Switzerland; 2 Swiss Finance Institute, c/o University of Geneva, Geneva, Switzerland; University of Zaragoza, Spain

## Abstract

We identify and explain the mechanisms that account for the emergence of fairness preferences and altruistic punishment in voluntary contribution mechanisms by combining an evolutionary perspective together with an expected utility model. We aim at filling a gap between the literature on the theory of evolution applied to cooperation and punishment, and the empirical findings from experimental economics. The approach is motivated by previous findings on other-regarding behavior, the co-evolution of culture, genes and social norms, as well as bounded rationality. Our first result reveals the emergence of two distinct evolutionary regimes that force agents to converge either to a defection state or to a state of coordination, depending on the predominant set of self- or other-regarding preferences. Our second result indicates that subjects in laboratory experiments of public goods games with punishment coordinate and punish defectors as a result of an aversion against disadvantageous inequitable outcomes. Our third finding identifies disadvantageous inequity aversion as evolutionary dominant and stable in a heterogeneous population of agents endowed initially only with purely self-regarding preferences. We validate our model using previously obtained results from three independently conducted experiments of public goods games with punishment.

## Introduction

Why do we maintain moral attitudes, display other-regarding behavior, have a distaste for unfairness, act prosocially and, at times, even behave altruistically towards others? How is this behavior compatible with the predominant theories of rational choice, selfish utility maximization and, in particular, with Darwin's principle of the survival of the fittest? This article presents an evolutionary utility framework of fairness, altruistic punishment and cooperation. It develops quantitative arguments supporting the hypothesis that the key to understanding the ostensibly mysterious patterns of human behavior is deeply rooted in our evolutionary history.

The analysis of our expected utility model, in combination with the underlying evolutionary dynamics, allows us to identify and explain the origin and the emergence of other-regarding preferences and, ultimately, enables us to quantitatively explain the degree of altruistic punishment that is observed in lab experiments. As a result, our approach complements and extends other utility frameworks, e.g. the Fehr Schmidt model [1], Bolton/Ockenfels [2] and Rabin [3], as well as existing evolutionary models linked to economics [4]–[10], by combining both perspectives in order to explain prosocial behavior. Unlike other approaches, our model does not assume ex ante the existence of other-regarding preferences, but instead demonstrates their co-evolutionary emergence along with the emergence of altruistic punishment behavior. The design of our model is inspired by previous findings about the co-evolution of culture, norms and genes, the effect of other-regarding behavior as well as bounded rationality. We motivate our model by the psychological predisposition of individuals to maximize their expected utility together with subliminal disposition to follow social norms [11]–[15]. Both mechanisms are closely related in the process of gene-culture co-evolution.

The following section 0 describes the model in detail and explains the interplay of agents who maximize their expected utility under the effects of natural selection and competitive evolutionary dynamics. Then, section 0.0 presents empirical tests of the theory. Section 0.0 establishes the evolutionary dominance of the specific other-regarding preference in the form of disadvantageous inequity aversion. Section 0.0 concludes.

## The Model

### 1 General framework

We take an evolutionary approach as a starting point to construct our model. The fitness of an agent is considered to be equivalent to her realized cumulative payoff relative to that of other agents, i.e. to the monetary units (MU) that the agent gains over time relative to the average over all other agents. Each agent 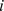 is characterized by one or multiple traits. The traits of an agent determine her behavior and correspond to a pure strategy denoted by 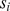. Traits are passed on as fitness weighted values to the offspring in the process of evolutionary reproduction. The population is determined by the set of pure strategies 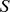. In an evolutionary competitive environment, agents are subject to natural selection which affects their viability and fertility. While viability selection accounts for removing poor performing agents from the population, fertility selection enables more successful agents to spread and to promote their genetic and cultural heritage in the population. This process corresponds to the standard evolutionary challenge of survival and reproduction. Following the Darwinian principle of the survival of the fittest, both selection mechanisms are defined relative to the environment of an agent. This means that the fitness of an agent is determined relative to the performance of the remaining population that she is exposed to and interacts with. In an evolutionary environment, the success of an agent and of her strategies defines the fitness of the agent and thus determines the proportional change of the strategies (traits) in the population over time.

The set of strategies 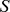 that characterizes a population of agents is specified by a probability measure 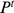 that quantifies the frequencies of the single strategies 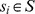 in the population at time 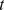. In the two player case, the payoff of an agent who plays a pure strategy 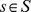 against another agent who plays the pure strategy 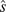 is denoted by 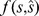. Both, 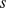 and 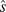 are defined in the continuous strategy space 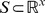 (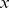 depends on the game; in the public good game studied here, 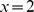 corresponds to the decision on the contribution level 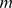 and on the chosen propensity 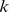 to punish for each agent). For the 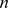-player case, the average payoff of an agent who plays a strategy 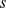 at time 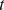 against a population characterized by the probability measure 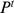 over the strategy space 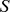 is defined by
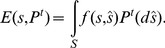
(1)The total average payoff of the entire population at time 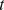 is defined by
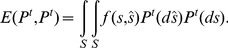
(2)The success of a strategy 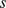 is given by the difference of equations (1) and (2) as shown e.g. in [16]–[18]:
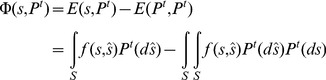
(3)The dynamics of the frequency of a specific strategy 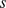 in the population is defined by the ordinary differential equation
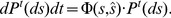
(4)The function 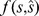 in equations (1–3) reflects the underlying payoff structure of the analyzed game. In the context of this paper, 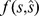 represents the payoff function of a public goods game with punishment.

### 2 The public good games with punishment

In the following, we model the behavior of agents playing a standard one-shot-interaction public goods game with punishment as presented in [19]–[21]. Agents are pooled in groups of size 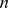. Each agent 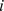 is characterized by a strategy 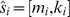 that is defined by two traits. The first trait 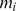 corresponds to the amount of MUs an agent contributes to the common group project (the public good) and thus reflects the agent's willingness to cooperate. The second trait 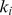 reflects the agent's propensity to punish defectors in the group.

The general procedure of the public goods game with punishment is as follows. In the first stage of the game, agent 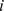 contributes 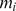 monetary units (MUs) to a common public good which yields a return of 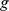 MUs per invested MU. The return from the public good is equally redistributed among the 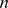 group members. Agents then learn about the contributions of the other group members. In a second stage, they are provided with the opportunity to punish other group members. Punishment comes in the form of additional costs for both the punisher as well as the punished agent: for each MU spent by the punisher, the return that the punished agent obtained from the public goods game is reduced by 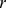 MUs. Given the one-shot-interaction characteristic of the game, punishment does not result in a direct or indirect material benefit and is often considered in the literature to be an altruistic act.

### 3 Modeling assumptions

**Figure 1:**
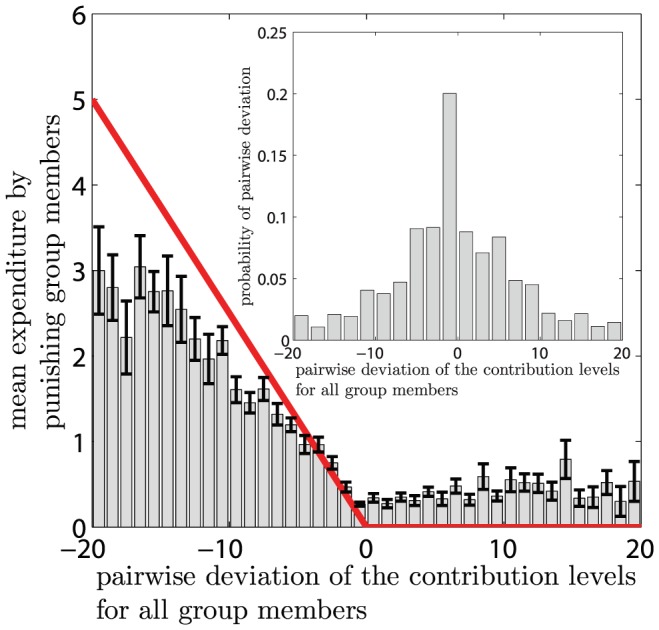
Mean expenditure of a given punishing member as a function of the deviation between her contribution and that of the punished member, for all pairs of subjects within a group, as reported empirically [19]–[21]. The error bars indicate the standard error around the mean. The straight line crossing zero with a slope of 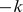 shows the average decision rule for punishment. The anomalous punishment of cooperators, corresponding to the positive range along the horizontal axis, is neglected in our model. The inset shows the relative frequency of the pairwise deviations.

We make the following assumptions about the behavior of agents and the evolutionary environment:

- Agents are assumed to be self-interested and to act rationally given their available information and computational capabilities [22]–[25]. In particular, agents are involved in one-shot interactions only and have no ex-ante information about the others' actions at the time they take their decisions. Thus, other agents' past actions (history) will not affect the agent's current or future decisions. This corresponds to the so-called stranger treatment in the experiments that we analyze below. This can also be called the strong mixing regime with rapid memory loss. A perhaps more convincing interpretation of this framework is in terms of a coarse-grained description of the multiple interactions and feedbacks between agents within groups that act over time scale of generations. In this interpretation, the unit time step is roughly commensurate with the agent lifetime. Then, other agents' past actions and history occur essentially within each time step but not beyond, justifying the model assumption.
- Agent 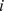 is assumed to punish agent 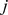 according to a function that is linearly increasing with the negative deviation between 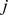
's and 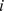
's contributions. Specifically, if 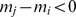, agent 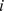 punishes agent 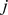 by spending 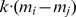 MUs, while 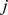 suffers a loss of 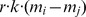 MUs. We assume this linear dependency because it can frequently be observed in experiments conducted in the western cultural area [19]–[21], [26], [27]. Figure 1 illustrates this behavioral pattern for data obtained in three public goods games [19]–[21].
- The factor 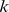 describes the agents' propensity to punish negative deviators. 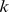 is assumed to be a common trait or a norm that is shared by all agents within a homogeneous population. It reflects the subjects' genetically and culturally encoded behavior to react to actions that are e.g. perceived as being unfair. The interplay of punishment and evolutionary dynamics (adaptation, selection, cross-over and mutation) over hundreds of thousands of years caused the convergence of a previously diverse set of behavioral patterns. This process ultimately led to a common set of behavioral traits which are shared among directly- or indirectly-related and -interacting individuals, e.g. groups originating from the same cultural area. Vice-versa, the prevalent set of behavioral traits determined the anticipated expectations about the behavior of individuals from the same cultural and genetic background. Punishment thus provided the basis for the emergence and manifestation of traits and (social) norms, while simultaneously punishment itself got frequently established as a common trait and norm. In conclusion, humans and our ancestors have converged and evolved to this common norm-enforcing feedback mechanism over hundreds of thousands of years as a result of gene-culture co-evolutionary processes [28]–[30]. The subjects' psychological predispositions to render these encoded norms effective ultimately results in the focal action that is observed as a direct and immediate harm towards negative deviators or it acts as a hidden deterrence [11]. Today, lab experiments and field studies such as those of [19]–[21], [31], [32] allow one to sample and observe the statistically stationary characteristics of the common propensity to punish 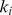 from subjects originating from a similar cultural background.
- The population of agents is subject to evolutionary dynamics in the form of selection, cross-over and mutation. These three mechanisms affect the viability and fertility of an agent. Viability selection induces a minimal survival condition in the form of a fixed lower value of consumption 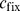. This value reflects the basic requirements of an agent, i.e. it defines a lower limit that an agent needs to consume per unit of time in order to survive. 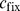 thus repeatedly absorbs a fraction of the agents' fitness value. Fertility selection accounts for the selection of successful genotypes, i.e. strategies, as opposed to unsuccessful ones. Agents can spread their strategies in the population proportionally to their fitness, e.g. by producing more offsprings. The relative change of the frequency of a trait, i.e. a strategy, is determined by the average success of that trait with respect to the average success of the remaining traits in the population. Cross-over, i.e. the reproduction by mating of two or more agents, accounts for the convergence of the present strategies in the population towards those strategies that are carried by more successful agents. In contrast, mutation induces an additional heterogeneity to the agents' strategy pool and allows the population to explore further the potential strategy space. This ensures that a population of agents is always heterogeneous with respect to the strategies, i.e. 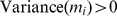 and 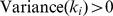.

### 4 Utility formulation of the public goods game model

We first formulate a fitness model assuming complete information. The profit and loss (P&L) of an agent 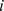 that plays a public goods game with punishment is determined by the payoff from the game minus the costs of punishing and being punished and minus the contributed effort:
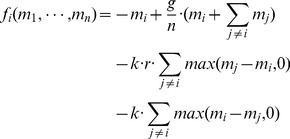
(5)The first term in the right hand side of equation (5), i.e. 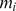, corresponds to the contribution of agent 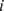 to the public good. The second term represents the return from the public good. The third and fourth terms quantify the costs of being punished by others and punishing others, respectively. The number of agents in the group is denoted by 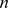, the return from the public good is 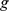 per invested MU, and 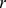 corresponds to the punishment efficiency factor.

Analogously, the P&L of the remaining agents 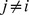 can be written as
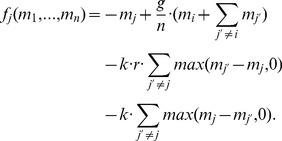
(6)


By writing the relative fitness of an agent according to equation (3) in the form of an evolutionary measure of success, we obtain the fitness of agent 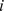 as the sum of the experienced payoff differences between the own monetary payoff 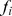 and the monetary payoff of the remaining individual group members 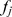:
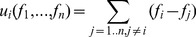
(7)The relative fitness of an agent is thus obtained by putting the payoff of agent 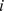 in relation to the payoff of the remaining population. This form of the fitness describes a population of agents that is exposed to evolutionary dynamics as presented in section 0.1: Positive values of 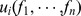 are desirable, because they are associated with a higher fertility and a lower mortality. Negative values of 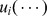 should be avoided in order to prevent the evolutionary extinction of the own traits.

In our attempt to combine preferences, expected utility and evolution, we should be careful and clarify what is meant by fitness and by utility. It is customary to distinguish fitness from utility, the former being proportional to material payoffs as defined above (eventually controlling the number and quality of offsprings), while utility refers to preferences. In full generality, it may be that preferences differ from payoffs, as when utility depends on the presence of salient unchosen alternatives that do not have any impact on the payoffs [9]. This distinction may lead to a fruitful strategy to understand how evolution processes may lead to apparent dysfunctional preferences [8] and contribute to explain the nature of our utility functions [9]. However, since our purpose is limited to provide a generalization of utility approaches of [1], [2] and [3] to explain the experimental results of three independently conducted experiments of public goods games with punishment, we shall neglect the difference between fitness and preference. For our purpose, agents in our game as well as in the experiments are not exposed to salient unchosen alternatives, suggesting that we can neglect the distinction between fitness and utility within our framework. The issue of fitness versus utility falls within the bigger question of the so-called “adaptationist program”, which has dominated evolutionary thinking, and its possible caveats have been insightfully presented by [33].

Henceforth, by substituting equations (5) and (6) into equation (7), we obtain what can be termed the evolutionary utility of an agent, given by the two-term function shown in equation (8) below. The first term of (8) corresponds to agent 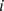
's utility gained from the payoff of the public goods game with punishment. The second term of equation (8) represents the payoff for each of the 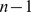 opponents indexed by 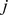. The total utility for agent 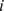 is defined by the sum of the differences between all combinations of 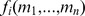 and 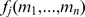 with 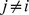:
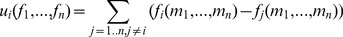
(8)


Consistent with utility theory (even in the presence of bounded rationality) and the underlying evolutionary dynamics, we assume that the agents seek to maximize their utility [22]. Obviously, the maximum of the utility function (8) can only be calculated in the hypothetical case of complete information about the others' contributions. However, information about the individual contributions 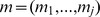 is not available ex ante, because agents decide about their contributions simultaneously. It follows that agents are required to make assumptions, i.e. to form their first-order beliefs, about the others' contributions. We model this by transforming the utility model in equation (8) into a subjective expected utility model.

Therefore, we introduce the subjective probability measure 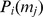 that represents agent 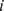
's (first-order) belief about the contributions of the other agents. 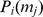 quantifies the likelihood as perceived by agent 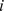 that another agent 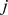 will contribute 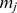 MUs. In the one-shot game version studied here, all agents 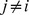 are indistinguishable from the point of view of an agent 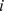, i.e., agent 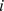 has no information on any preference, trait or specific characteristics of the other agents. Using 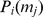, agent 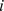 can form her expectation [34] about the average of the other agents' contributions:
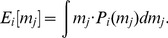
(9)Similarly to the propensity 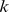 to punish, 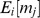 can be interpreted as the expected norm-conforming behavior of the population that has co-evolved, learned and internalized across time in a population of interacting agents.

The utility model defined in equation (8) is transformed into an expected utility model using the subjective expectations 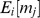. Rewriting 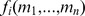 and 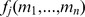 by replacing each value 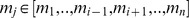 with agent 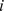
's subjective expectation 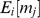 on 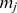 gives the following equations:
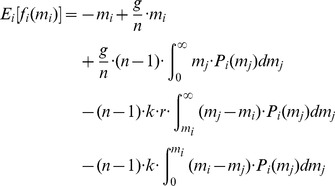
(10)

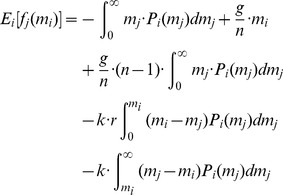
(11)Note that, in the formation of the expectation by agent 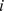 of the others' utility functions, agent 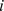
's own contribution 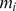 is obviously known to her, hence the term 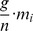 appears without averaging.

As in the case of complete information, agents seek to maximize their relative fitness, i.e. the sum of the differences between their own P&L, 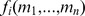, and the others' P&L. Putting all this together, we obtain the evolutionary expected utility function 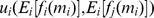 of agent 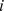 as shown in equation (12).
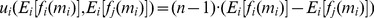
(12)


We start our analysis by a classical utility optimization problem. Agents maximize 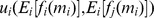 with respect to their contribution 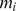:
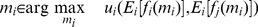
(13)The first order condition of problem (13) reads
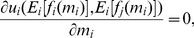
(14)with
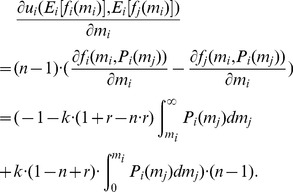
(15)


The second-order condition for a local maximum of (13) holds for any reasonable assignment of the problem parameters, i.e.
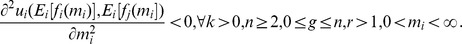
The cumulative distribution function of the contributions 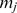 of the other agents, as anticipated by agent 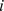, is defined by 
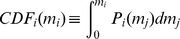
. The term 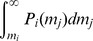 in equation (15) corresponds to the survival function of the subjective expected distribution of contributions in the population:
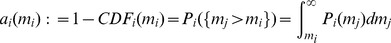
(16)Substituting 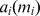 as defined in equation (16) into equation (15) yields:
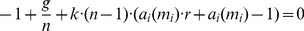
(17)
Equation (17) describes a functional relation between the predetermined parameters of the public goods game, i.e. the group size 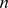, the project return factor 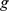 and the punishment efficiency 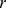, as well as the variable traits of agent 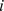, i.e. the propensity 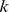 to punish and her subjective expectation (first-order belief) about the fraction 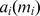 of her group fellows who contribute more than her own contribution 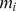.

As we are interested in the agents' evolutionary optimal punishment behavior, we solve equation (17) for 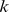 and obtain:
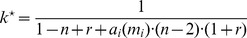
(18)

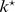
 depends on 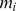 via agent 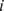
's subjective (first-order) belief embodied in 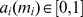 that the other agents will contribute more than herself. Thus, 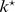 can be interpreted as the value that makes agent 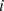 better off not to deviate negatively or positively from her willingness to contribute 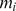 MUs to the public good, given she believes a number of 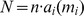 of other group fellows contribute more than her own contribution 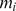.

In the following subsection, we consider evolutionary dynamics in our model.

### 5 Evolutionary dynamics of the level of cooperation 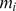 as function of the propensity to punish 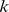


The evolutionary dynamics of agents, who face a social dilemma situation in the form of a public goods game with punishment, can be described in terms of the agents' P&L as a function of the deviation in the contribution level 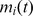 and of the population's common propensity to punish 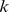. If agent 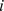 starts to deviate from her current level of cooperation 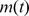 by a value of 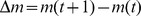, the absolute change of the P&L for the agent as a function of 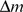 and 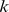 is defined as follows:
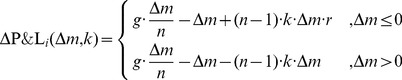
(19)These expressions assume that the deviation affects a single agent, who deviates from the norm. Hence, the agent is punished by (resp. punishes) all other 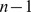 agents for 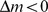 (resp. 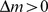).

The deviation of agent 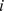 by 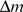 affects not only her own P&L, but also the P&L of the remaining agents 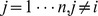. The absolute change of the P&L of the remaining population as a function of 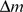 and 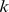 reads
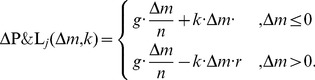
(20)


Putting equations (19) and (20) together with
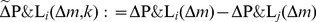
(21)yields the relative change of the P&L of agent 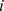 with respect to the remaining population:
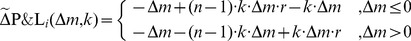
(22)


The form of equation (22) is equivalent to the relative measure of success introduced in equations (3) and (7) with 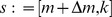. As introduced above, the realized P&L from the public goods game with punishment can be interpreted as the fitness of an agent in an evolutionary environment. The fitness, in turn, is associated with the rate of fertility, i.e. the fitter an agent becomes, the more genetically related offsprings she produces. In this way, traits of agents with a higher realized P&L value tend to spread and to end up dominating the population over time. It thus holds that the traits 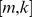 in the population move with time towards values 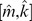 of a subpopulation that on average achieves a higher mean P&L than the average mean P&L of the entire population.

The corresponding replicator dynamics are
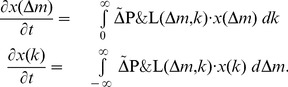
(23)with 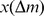 and 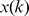 being respectively the proportion of agents deviating by 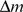 and with a propensity to punish 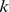. Extending the previous reasoning, expressions (23) now include the occurrence of deviations from the optimal (for the community) propensity to punish, which may result for instance from random mutations. The dynamics for the expected group average, 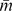 and 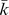, are accordingly defined by
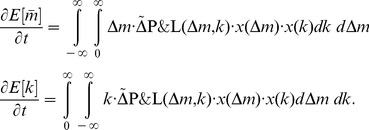
(24)


The sensitivity of 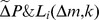 given by (22) with respect to the relative change of 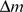 is defined by the partial derivative
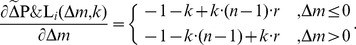
(25)


With the conditions that 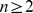 and 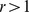, i.e. a game has always two or more players and punishment is less costly to the punisher than to the punished agent, it holds that, for 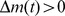, 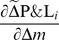 is always negative and, for 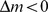, 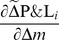 is always positive:
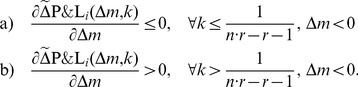
(26)This reveals the existence of two distinct evolutionary regimes that are separated by the bifurcation point at
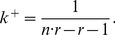
(27)

- *Defection*: For 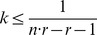 and 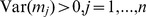, the linear P&L structure of the public goods game with punishment together with the replicator dynamics are responsible for 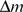 to become more negative over time. It intuitively follows that defection pays out, such that
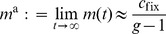
(28)results as the evolutionary stable behavior. Remember that each agent has a minimum cost of living defined by 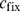. In order to meet this survival condition, the average minimum contribution of the population is constrained to values of 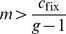.
- *Coordination*: For 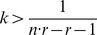, a heterogeneous population with 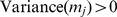 for 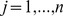 follows a dynamic that does not converge to a predetermined unique evolutionary attraction point but rather converges to an evolutionary stable set of strategies. As punishment is efficient in this regime, with 
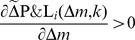
 for values of 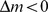, the social dilemma problem transforms into a coordination problem [1]. If punishment is efficient, the utility maximizing strategy is to contribute according to the expected contribution of the remaining group fellows, i.e. to contribute according to the first-order belief. Following Black's theorem, the best estimate for this strategy is the median value 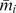 of the subjective probability measure 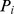 that is believed to characterize the contributions of the group fellows [12], [35]–[37]. The median value 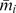 of the subjective probability distribution 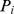 is defined by
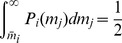
(29)Consequently
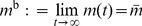
(30)results as an evolutionary stable behavior in the population.

**Figure 2:**
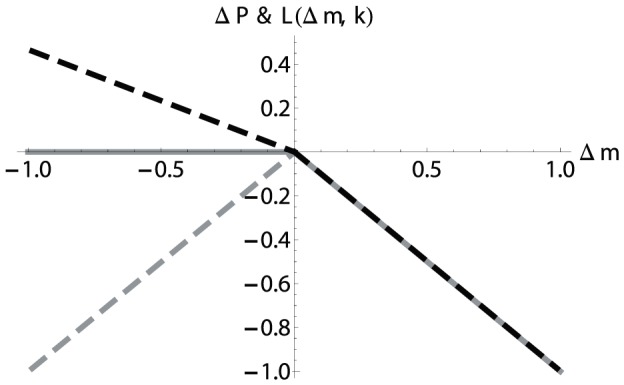
Sensitivity of 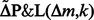 as a function of a relative change 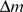 of the contributions for a group size of 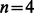, a punishment efficiency 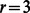 and a propensity to punish of 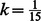 (black, dashed), 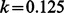 (grey) and 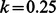 (grey dashed).

Figure 2 depicts the structure of equation (22) with a punishment efficiency factor 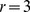 and a group size 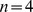 for 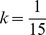 (black, dashed), 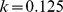 (grey) and 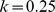 (grey, dashed).

The next subsection analyzes the identified evolutionary stable strategies (ESSs) for a population of agents that is either purely self-regarding and acting selfishly or a population of agents that incorporates other-regarding preferences in their decision process.

### 6 Coevolutionary dynamics of the propensity to punish 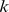 and the level of cooperation 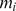 in the presence of self and other-regarding preferences

First, consider a population of purely self-regarding and selfish acting agents, i.e. agents who try to maximize their utility without e.g. taking into account specific preferences with respect to the P&L and the contributions of the remaining agents in the group. The preferences of self-regarding and selfish agents are simply characterized by the dislike of situations in which their P&L in current period 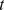 is less than their P&L in the previous period 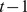. This implies that all agents in the population are satisfied if and only if the following expression is fulfilled
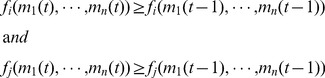
(31)for all 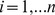 with 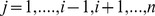. The functions 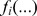 and 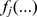 are defined in equation (5) and (6), respectively. Reducing the expression in (31) over the domain of reasonable values for the variables 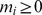, 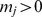, 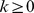, 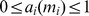, 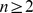, 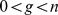 and 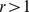 and solving it to the propensity to punish 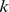 gives the following condition:
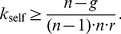
(32)The population of agents initially consists of uncooperative, non-punishers, i.e. 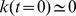 and 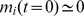 for 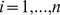. For all reasonable values of 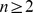, 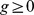 and 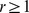, it holds that the desired propensity to punish of self-regarding agents is less than the bifurcation threshold 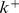, defined in equation (27):
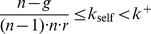
(33)With the population being initialized at 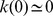 and the condition in equation (33), it follows that agents have an incentive to defect as can be inferred from equation (26a). In other words, agents have an incentive to contribute less than the amount contributed by the other group fellows. In general, agents have no ex-ante information about the others' contributions at the time they take the decision to contribute 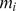 MUs. However, agents have beliefs about the others' contribution that is embodied in the subjective probability distribution 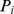. This allows them to form their expectations about the group average contribution as defined in equation (9). In terms of equation (16), “defecting” translates into a probability of one that all 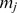 values are larger than the own contribution 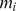, i.e. 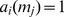. With 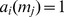, it follows that the optimal propensity to punish defined in equation (18) becomes
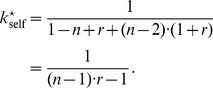
(34)which is exactly equivalent to the evolutionary threshold value of 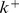 defined in equation (27). Plugging 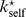 into equation (22) yields
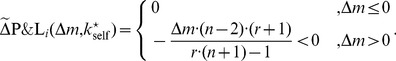
(35)Together with the replicator dynamics defined in equation (24), it follows that the population converges towards the evolutionary stable attraction point for 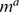 that is defined in equation (28). For the evolutionary dynamics of 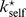, it holds that
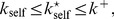
(36)i.e. the value range of the propensity to punish is restricted to the interval 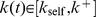. Thus, selfish and purely self-regarding agents are inevitably caught in the *defection* regime, as 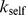 does not allow to overcome the bifurcation hurdle at 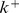. Consequently, the population converges towards the ESS that is defined by 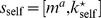.

Consider now a population of agents who display other-regarding behavior in the form of disadvantageous inequity aversion. In general, inequity aversion preferences relate the personal utility gained from a public good to the personal contributed effort. If an imbalance exists between the own contributed effort and the personally received payoff compared to the performed effort and the received payoff of other agents in the group, the outcome of the game is perceived as being inequitable or “unfair”. Disadvantageous inequity aversion implies that subjects only dislike situations in which the inequity is to their disadvantage. The payoff of an agent 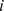, who plays a public goods game with punishment, is defined by equation (5) and the personal effort is equivalent to the contributed amount of MU 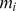. An agent with an aversion against disadvantageous inequitable outcomes thus does not like situations in which

- she contributes equally or more than her group fellows (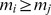) and receives a payoff that is smaller than the average utility received by the remaining group members (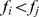) or
- she contributes more to the public good (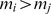) and, at the same time, receives a payoff that is smaller or equal to the remaining group's utility (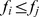).

By implication, the population of agents is satisfied if, and only if, at least one of the following three conditions is fulfilled for all 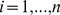 with 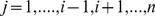:
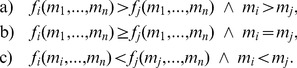
(37)


Expressing the above conditions (37) over the domain of eligible values for the variables 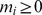, 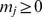, 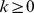, 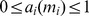, 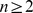, 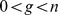 and 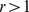 and solving them in terms of the propensity to punish 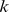 yields the following condition:
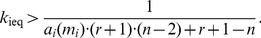
(38)For all values 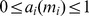, it holds that 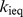 in condition (38) becomes larger than the evolutionary threshold 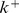 defined in equation (27) such that
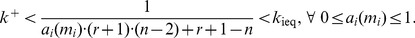
(39)Thus, agents are forced to switch from the *defection* regime into the *coordination* regime in order to satisfy their preferences. As described before, the best response strategy in the coordination regime regarding the level of cooperation 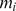 is to contribute according to the median value of the subjective probability distribution 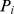. By the definition in equation (16), it follows that the median value 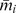 of 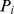 is equivalent to a value of 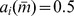. Plugging 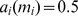 into equation (18) yields the following estimate for the optimal propensity to punish among disadvantageous inequity averse agents
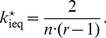
(40)From that fact that 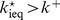, it follows that an evolutionary stable attraction point 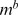 emerges, which has been defined in equations (29) and (30), respectively. Substituting equation (40) into equation (22) results in a 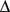
P&L profile that is determined by symmetrically downward sloping functions 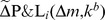 centered relative to the maximum at 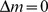 with
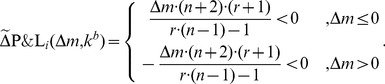
(41)The population of agents is thus able to maintain a stable level of cooperation at the median value 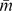 that is determined by the initial set of subjective expectations about the other contributions embodied in the distributions 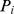.

In conclusion, a population of disadvantageous inequity averse agents converges to the ESS that is determined by 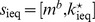.

Our first main result can be summarized as follows:

**Result 1:** *In the presence of standard Darwinian evolutionary dynamics, agents' traits (strategies) converge to evolutionary stable strategies, which results in a public goods game with punishment to be either characterized by defection (for weak punishment) or by coordination (for sufficient punishment). Purely self-regarding agents are inevitably caught in the defection regime while disadvantageous inequity averse agents are able to resolve the social dilemma by transforming it into a coordination problem.*

In the following section, we turn to the empirical validation of our model.

## Empirical Test of the Theory

In this section, we compare the predictions derived from our model with the empirical data obtained in three independently conducted lab experiments and validate our results against the empirical observations.

### 1 Description of the empirical data set

We analyze data from three public goods game experiments with punishment [19]–[21], which were carried out independently. In each experiment, groups of 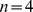 subjects played a two-stage public goods game: at the beginning of stage one, the contribution step, individuals were endowed with 20 monetary units (MUs). Subjects could decide on the amount 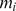 of MUs to contribute to the public good. The sum of all contributions was compounded by a factor of 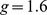 and subsequently redistributed in equal shares to all group members. Note that this results in a per capita gain of 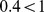 per contributed MU, which induced a distinct social dilemma component. In the second stage, the punishment step, subjects were informed about the contributions of their group mates. Subsequently, they could spend an additional fraction of their endowment to punish other group fellows. Each MU spent by the punisher caused a harm of approximately 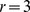 MUs to the punished subject. The punishment step in the first experiment was slightly different from the other two experiments: the punishment efficiency factor was determined based on the first stage payoff of the punished individual. However, it can be considered to be approximately equal to the factor 3 as in the remaining two experiments.

These two stages were played repetitively either in a stranger or a partner treatment. In the former, group members were reshuffled after each iteration to preserve the characteristics of one-shot interactions, i.e., to control for direct reciprocal effects. In the partner treatment, subjects played continuously with the same group members across all periods. The first experiment was composed of both a stranger and a partner treatment. Each of them were played for 10 periods. The second and third experiments included only a stranger treatment and were played for 6 and 10 iterations, respectively. In addition, the third experiment differed in the way information about the received punishment was revealed to the punished subjects. In the first one, the so-called observed treatment, subjects were informed immediately after the punishment stage about the costs of the received punishment, as in experiments one and two. In contrast, in the second treatment, the unobserved treatment, subjects were informed about the costs they had to bear for being punished only after the last period had been played. However, the results of both treatments were found not to be significantly different as the fear of punishment seems to be as effective as the punishment itself [21]. To obtain a sufficiently large sample size, we pool the observations from all treatments of the three experiments introduced above. The subject pool size amounts to a total of 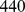 subjects.

### 2 Recovering the propensity to punish from the empirical data

The empirical propensity to punish can be calculated by taking the observed deviations 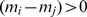 between subject 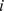 and 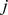 and the observed punishment from subject 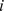 to 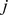, 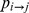. In this way, each pairwise interaction between two subjects provides a realization for the propensity to punish according to the formula
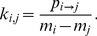
(42)With the set of all pairwise interactions, we construct the empirical distribution of the propensity to punish, by sampling all realized 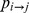 with their corresponding 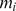 and 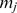.

As shown in the first section and also demonstrated in [38], the agents' propensity to punish can be interpreted as a norm-enforcing behavior that has co-evolved over tens and hundreds of thousands of years by gene-culture co-evolution along with the emergence of an aversion to disadvantageous inequitable outcome. The perception of fairness and the reaction to unfair behavior seems to be deeply rooted in our cultural and genetic heritage [28], [39], as experiments and field studies across different locations and cultural groups suggest [15], [31]. We thus consider the propensity to punish 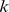 to be a constant on the evolutionary negligible short time-scale of the experiments. This can be substantiated by comparing the results of a two-sample Kolmogorov-Smirnov test between an empirical data set containing only data from the first period and the corresponding full-sample data set. The null hypothesis that the distributions of the two data sets of 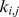 result from the same generating mechanism cannot be rejected (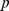-value equal to 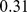). In all three experiments, the observed contributions 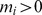 are approximately stable over time, as they do not converge towards full defection. Additionally, the standard deviation of the contributions is on average decreasing over time. Both of these measures indicate that the subjects in the experiments are in the “*coordination*” regime.

### 3 Validation of the model prediction for 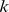


We validate the model by asking whether the ESS value 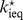 of the propensity to punish in the *coordination* regime given by equation (40) matches the empirically observed data. The group size 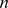 and punishment efficiency 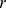 are known parameters in the experiments. The three public goods game experiments with punishment [19]–[21] have been performed with 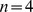 players and a punishment efficiency factor of 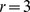, respectively. Plugging both values into equation (40) yields
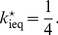
(43)As the value given by (43) is based on the assumption that subjects contribute according to the median value of their subjective probability distribution about the contributions of their group fellows, 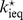 corresponds consequently to the median of the distribution of the values 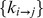 of the propensity to punish.

Remarkably, we find an exact match with the median value 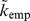 estimated from the empirical distribution of the 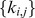 values, i.e. 
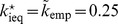
. The standard error of the median of the empirical data is 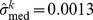. This corresponds to a one-standard error range given by 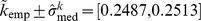. The corresponding 95% confidence intervals for the sample median values are 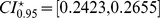, 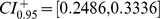, 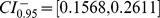 and 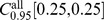. To estimate the confidence intervals we used a bootstrap t-method presented in [40].

The superscript on the 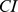 indicates the individual data sets:

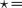
 [19]

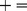
 [20]

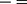
 [21]

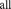
 = pooled data set of all three experiments.

This remarkable agreement between theory and empirical data suggests that subjects act according to the optimization problem defined in (13) and that their punishment behavior is dominated by disadvantageous inequity aversion preferences defined in equation (37). Again, we argue that in this specific setup the focal action to punish negative deviators by spending roughly a fourth of the negative deviation has emerged as the result of the human's psychological predisposition to render effective the culturally and genetically internalized norms [11], [38]. In this case, these norms are described by disadvantageous inequity aversion. We can now state our second main result:

**Result 2**: *The level of altruistic punishment that subjects exhibit in public goods game experiments can be explained by a simple aversion to disadvantageous inequitable outcomes together with the individual maximization of the expected utility defined in* *equation (13)* *and the presence of evolutionary dynamics.*

**Figure 3:**
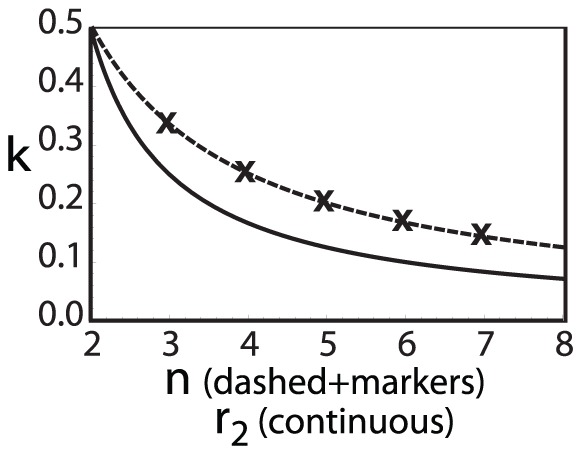
Propensity to punish as a function of the punishment efficiency 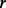 (continuous line) for a fixed group size 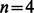 and as a function of the group size 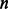 (dashed line with cross markers) for a fixed 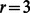.

The dependence of the optimal propensity to punish 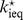 defined in equation (40) on the group size 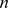 and the punishment efficiency factor 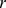 is plotted in figure 3. This predicts the potential propensity to punish that should be observed in experiments with differing configurations. In particular, the larger the punishment efficiency 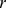 and the group size 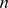, the smaller becomes the optimal propensity to punish. To validate these predictions additional experiments with different groups sizes and punishment efficiency factors have to be performed in future research.

The following section analyzes the co-evolutionary dynamics of agents with disadvantageous inequity aversion compared to agents with purely self-regarding and selfish behavior in a heterogeneous population.

## Evolutionary Dominance of Other-Regarding Preferences

The results and findings presented in the previous two sections inevitably raise the question about the evolutionary stability and dominance of other-regarding compared to self-regarding preferences. Are agents with other-regarding behavior able to invade a population of initially selfish and self-regarding agents? Can the required conditions for the emergence of altruistic punishment spread in a population of agents that is facing a competitive resource limited environment as described by our model? Is disadvantageous inequity aversion the predominant strategy in a population of agents who face a social dilemma situation that provides the opportunity to punish? This section addresses these questions by providing an analysis of the co-evolutionary dynamics that are at play in a heterogeneous population consisting of a mixture of disadvantageous inequity averse agents and purely self-regarding and selfish-acting agents.

A system that is subject to evolutionary forces is characterized and determined by selection, cross-over and mutation processes. Consequently, the birth and death of agents induce multifaceted and complex co-evolutionary dynamics that are contingent on the states and path dependencies of the individual actors in the system. In view of this complexity, this section presents a simplified but conclusive analytical representation of the system's dynamics and properties. This is achieved by reducing the assumed heterogeneity in the system and by considering only two groups and types of agents, respectively. An extensive numerical analysis of a population of agents playing a public goods game with punishment that takes into account the full heterogeneity and the full set of evolutionary dynamics and path dependencies is presented in the companion article [38].

### 1 Conditions for evolutionary dominance

Let us write the evolutionary success of a homogeneous group 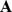 of agents with size 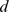 playing strategy 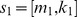 that competes with a homogeneous group 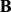 of size 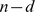 with agents playing strategy 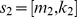. Using equation (3) and the P&L structure of the public goods game with punishment defined in the equations (5,6), we obtain
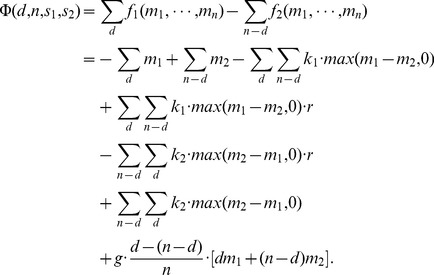
(44)Expression (44) can be rewritten by forming the expectations with respect to the evolutionary success 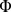 and assuming that group 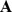 randomly varies in the contribution behavior of its agents. Therefore, the contribution 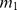 (per agent) of group 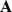 is assumed to deviate from the contribution 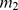 (per agent) of group 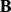. The total expected deviation of group 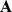 is defined by 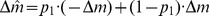 where 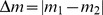. Each of the two groups is assumed to be intrinsically homogeneous but differs from each other, not only in the expected contributions, but also with respect to the punishment behavior, i.e. 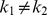. Agents in group 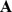 are characterized by the propensity to punish 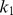, while group 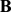 exhibits a propensity to punish that corresponds to 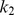. The average evolutionary success (or failure) of group 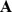 with 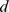 members who deviate negatively with a given probability 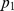 or positively with the probability 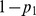 by a value 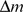 from the contribution 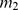 of group 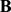 which has a total of 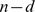 members is given by
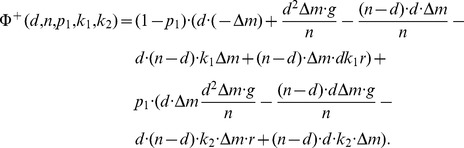
(45)The measure 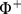 defines a relation between the relative difference of the P&L of group 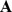 versus that of group 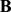. It thus reflects the evolutionary success or failure of the two competing groups over time. An expected deviation of group 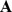 by a value of 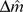 affects 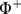 to become either positive or negative. Depending on the sign of 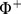, either the strategies of group 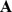 start to dominate the population (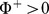) or alternatively, if 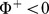, the strategies of group 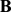 spread and dominate in the population.

### 2 Evolutionary dominance of disadvantageous inequity averse agents

Consider a population of size 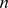 that initially consists only of purely self-regarding and selfish acting agents. This homogeneous population is assumed to be in an evolutionary equilibrium state. As identified in the previous sections, self-regarding agents play the ESS 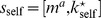 with
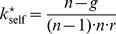
and
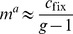
as given by the equations (32) and (28). Replacing one agent in this population by a disadvantageous inequity averse agent leads to a heterogeneous population that consists of two homogeneous subgroups. In the following, we analyze the co-evolutionary dynamics of this heterogenous population of agents that is composed of a group 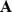 with size 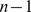 of purely self-regarding agents and a group 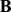 with a single disadvantageous inequity averse agent corresponding to size 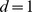.

In contrast to the self-regarding agents, disadvantageous inequity averse agents play the ESS given by 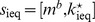 with expression (40) for
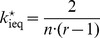
and
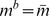
as defined in equations (40) and (30).

We need now to specify the punishment propensities 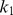 and 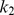 that enter into expression (45). For this, we take the view that agents are boundedly rational and choose their punishment propensity using a model of others based on homophily or, more precisely, a theory of minds that attribute to others one's own inclinations. This amounts to choosing 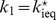 given by (40), 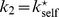 given by (34). Reporting these expressions together with 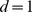 into equation (45), we obtain
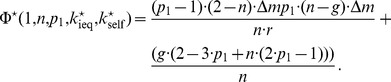
(46)


The logically consistent relation between the evolutionary success or failure, viewed either from the perspective of group 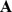 or from group 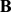, reads:
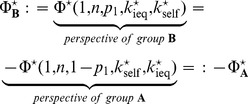
(47)If 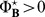, group 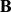 that initially consists of a single disadvantageous inequity averse agent, outperforms group 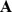 that has 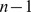 members of self-regarding agents. Consequently, the strategy 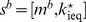 spreads in the population. In contrast, if 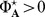, group 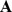 becomes predominant and strategy 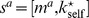 spreads in the population. The resulting condition for the disadvantageous inequity aversion trait to become dominant is defined by
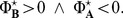
(48)


**Figure 4:**
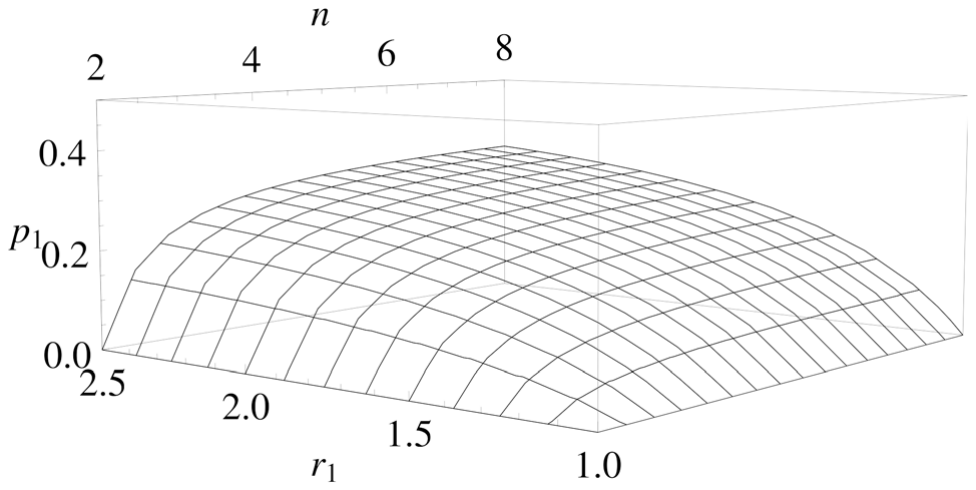
Minimum probability threshold 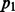 given by expression (49), above which a single disadvantageous inequity averse agent can invade a population of selfish agents by deviating from the contribution of the selfish agents with 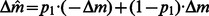.

Reducing condition (48) over the set of reasonable parameter values with 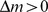, 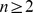, 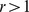 and 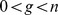 reveals that 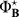 becomes positive if the probability 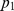 for deviating negatively falls into the range
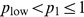
(49)with
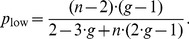
(50)
Figure 4 shows the surface defined by expression (49) for 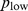 as a function of 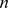 and 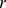 in the range 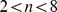 and 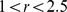. The domain above the surface corresponds to 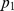 values for which a single disadvantageous inequity averse agent can invade a population of selfish agents by deviating from the contribution of the selfish agents.

A scenario with a population consisting of 4 agents with 3 agents being self-regarding and one agent being disadvantageous inequity averse, playing a public goods game with a per capita return of 0.4 MUs per invested MU, i.e. 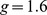, results in a 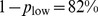 chance for the single disadvantageous inequity averse agent to outperform at each period.

For all reasonable parameter values, 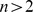 and 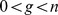, the lower bound 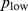 is always smaller than 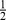. This means that the probability for the disadvantageous inequity averse agent to invade the population of selfish agents over time is always larger than one-half. The range of 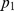 defined by equation (49) shows that, if the single disadvantageous inequity averse agent in group 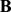 deviates on average by a negative value, i.e. 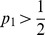, from the contribution 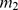 of the selfish agents (group 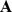), she always wins since 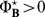.

Such a single agent can win even though she may be strongly out-numbered by the 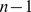 selfish agents who tend to defect, because the minimum required consumption 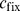 per period forces the population to contribute on average at least an amount of
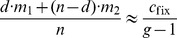
MUs in order not to go extinct.

On the other hand, if the single disadvantageous inequity averse agent contributes on average more than the group of self-regarding and selfish agents, it must hold that
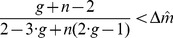
(51)in order for that agent to have a larger P&L than the self-regarding agents of group 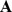. Coming along with the condition 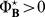, the disadvantageous inequity averse agent in group 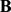 can be thought of as being more fertile than the self-regarding agents of group 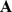, which results in 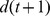 being larger than 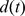 over time. In addition, with an increasing number 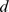 of agents in group 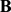 and, consequently, a decreasing number 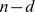 of agents in group 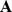, the lower limit for 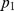 declines until it becomes zero for 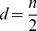. This means that, as soon as half of the total population consists of disadvantageous inequity averse agents, the self-regarding and selfish agents are doomed, as the probability for group 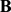 to take over the entire population becomes 1 independent of their contribution decisions. These results are similar to those obtained by [41], [42] on the “repeated prisonner's dilemma” game and variants, for which larger minimum usage frequencies are needed to stabilize strategies with lower efficiency or payoffs.

In summary, for arbitrary initial conditions, we have established that disadvantageous inequity averse preferences and the corresponding ESS 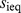 have significantly more than 50% chance of spreading in the population. At large times and for finite populations, in the presence of a larger than 50% probability to grow their relative population (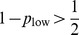), the population of the disadvantageous inequity averse agents will with probability one reach half the total population, at which point they invade with certainty the whole population due to their self-reinforcing advantage explained above. This can be summarized by the following set of inequalities:
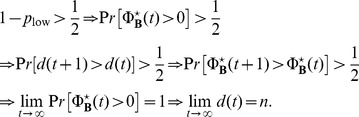
(52)


In conclusion, our third main result can be summarized as follows:

**Result 3**: *On long enough time scales, disadvantageous inequity averse preferences always invade and dominate pure self-regarding and selfish preferences in an evolutionary system.*

## Discussion

Prosocial behavior in humans has been studied in many laboratory experiments throughout the world. One key finding is the evidence for altruistic punishment behavior in humans, i.e. the punishment of non-cooperators and norm violators at own costs without direct or indirect material benefit [20], [21], [27], [43]–[49]. To allow for this pro-social behavior that is often marked as “irrational”, researchers shifted from purely self-regarding assumptions to theories that incorporated other-regarding preferences [50]. In particular, analytical frameworks of fairness, reciprocity and cooperation have been formulated that combine individual utility maximization with inequality and inequity aversion [1]–[3], [51]–[54]. These frameworks have a descriptive character and aim at reproducing the observed empirical behavior either quantitatively or using stylized facts. In this way, results from experimental economics have been rationalized and aligned with the predominant rational choice theory of pure self-interest.

Besides these equilibrium-based and time-independent utility theories, a second class of models emerged that focuses on the evolutionary origin of altruistic punishment and cooperation [55]–[62]. These models are often motivated from a biological perspective including arguments from evolutionary psychology, anthropology and sociology. Although the emergence of pro-social behavior in settings which are subject to material self-interest seems to contradict rational choice theory and the principle of the survival of the fittest, it is possible to show that cooperation and altruistic punishment can emerge and can be sustained in competitive, resource-limited environments [38], [63]–[66]. Let us also mention studies of spatial versions of public good games with punishment [67], [68], which have found that spatially evolving segregation between different cooperator and non-cooperator types may favor the emergence of cooperation. Contrary to the descriptive utility frameworks the evolutionary approaches aim at providing insight into the generating mechanisms of altruistic punishment and cooperation on an different level of analysis, and therefore are vague on what the exact nature of our social preferences should be. In the majority of cases, this is done by reproducing stylized facts rather than providing an external validity of the empirically observed behavior as it is the case for most of the descriptive utility frameworks.

The lack of a close connection between the evolutionary literature on cooperation and altruistic punishment and the experimental literature has led to intense discussion and diverse interpretations on what the experimental results show and do not show [69]–[74]. This paper has tried to fill the gap between the evolutionary theoretical literature on cooperation and punishment and the empirical findings from experimental economics by combining both worlds: an expected utility framework that allows for standard evolutionary dynamics in the form of adaptation and selection. In particular, we showed that the interplay of natural selection and adaptation by selfish utility maximization inevitably results in the evolutionary dominance of other-regarding preferences in the form of disadvantageous inequity aversion when competing with pure self-regarding behavior. The term “disadvantageous” implies a relaxation from the concept of inequity aversion and fairness preferences: Subjects only dislike situations in which the inequity is to their disadvantage. Consequently, no a priori stipulated modeling assumptions about altruistic and self-discriminating behavior are embodied. The aversion against inequitable outcomes causes altruistic punishment behavior to emerge, even in social dilemma situations that are subject to material self-interest. We have argued that the bare individual survival needs of our ancestors induced an inherent predisposition to unfairness aversion that persists in our behavior up to this day.

This argument might sound farfetched given that human beings are probably the most successful species in eluding or manipulating natural selection by continuous enhancing, e.g., via improvements of health care and medical engineering. However, at the same time, our cultural evolution developed higher, more abstract levels of selection mechanisms that operate e.g. as monetary, bargaining and market competition, and led to hierarchical structures of power and of social standing. In other words, the natural selection that was previously affecting and operating on our hunter-gatherer ancestors has substantially been replaced in our modern societies by social institutions, most notably by the advent of money and the measures of economic power. Our primal instinct to unfairness aversion is still subliminally active and can be triggered by this high-order social and cultural selection mechanisms. In consequence, the corresponding reactions to unfair behavior can be observed today even though we are in most situations not directly affected in our biological viability.

Previous works on economic theories about fairness, altruistic punishment and cooperation in voluntary contribution situations have systematically underestimated the importance of evolutionary dynamics and in particular the role of natural selection for the emergence of prosocial behavior and fairness preferences. We have combined an evolutionary approach together with an expected utility model to identify and explain the mechanisms that account for the emergence of fairness preferences and altruistic punishment. In particular, we designed an evolutionary utility model that allowed us to calculate an optimal strategy profile for the level of punishment in public goods games, depending on the fairness preferences of the agents in the population.

We considered two specific types of agents: (1) purely self-regarding agents and (2) agents who are disadvantageous inequity averse. We find that the evolutionary optimal strategy profile of disadvantageous inequity averse agents matches the behavior of subjects in the experiments and explains quantitatively the observed level of altruistic punishment without adjustable parameters. Our results imply that subjects show a strong predisposition for disadvantageous inequity aversion which, in turn, seems to be the driving force behind the observed altruistic punishment behavior. Finally, we showed that disadvantageous inequity aversion is an evolutionary dominant and stable strategy when compared to the pure self-regarding behavior, in a heterogeneous population of agents. Our theory offers new predictions that are testable by running future experiments with different numbers of subjects, modified payoff levels or a varied efficiency of the punishment.

In conclusion, we believe that path-dependent evolutionary processes, together with the self-organizational aspects of individual utility maximization, provide an important explanatory basis for the emergence of cooperation, altruism and prosocial behavior in general. Future research on social preferences should therefore include and focus on the time dimension and the evolutionary dependencies as well as the self-organizational capabilities of many social system.
